# Effects of Quinoa (*Chenopodium quinoa* Willd.) Consumption on Markers of CVD Risk

**DOI:** 10.3390/nu10060777

**Published:** 2018-06-16

**Authors:** Liangkui Li, Georg Lietz, Wendy Bal, Anthony Watson, Ben Morfey, Chris Seal

**Affiliations:** 1 Human Nutrition Research Centre, Institute of Cellular Medicine, Faculty of Medical Sciences, Newcastle University, Newcastle upon Tyne NE2 4HH, UK; Liangkui826@outlook.com (L.L.); Georg.Lietz@ncl.ac.uk (G.L.); Anthony.Watson@ncl.ac.uk (A.W.); Ben.Morfey1@ncl.ac.uk (B.M.); 2 Human Nutrition Research Centre, School of Natural and Environmental Sciences, Faculty of Science, Agriculture and Engineering, Newcastle University, Newcastle upon Tyne NE1 7RU, UK; Wendy.Bal@ncl.ac.uk

**Keywords:** quinoa, whole grain, CVD risk, continuous glucose monitoring

## Abstract

A number of epidemiological studies have suggested that diets rich in whole grains are linked to lower cardiovascular disease (CVD) risk and mortality. Quinoa, a pseudo-cereal, is included in the “whole grain” category but the effects of quinoa consumption in humans is not widely studied. Our aim was to undertake a dietary intervention study to investigate the effects of daily consumption of quinoa-enriched bread (providing 20 g quinoa flour) on CVD risk markers compared with a 100% refined wheat bread control. Thirty-seven healthy overweight men (35–70 years, body mass index >25 kg/m^2^) completed a 4-week cross-over intervention, separated by a 4-week washout period. Fasting blood samples were collected at the beginning and end of each intervention period. Continuous glucose monitoring was undertaken at the end of each intervention period. After 4 weeks of intervention, blood glucose and low density lipoprotein (LDL) cholesterol were significantly lower than baseline in both groups but there was no difference between quinoa and control. Anthropometric measures and other blood metabolites were not different between the two treatments. The cumulative area under the blood glucose curve for the last 4 days of the quinoa intervention tended to be lower than the first 4 days of wash-out (*p* = 0.054), and was significantly lower than the corresponding period of the wheat treatment (*p* = 0.039). In conclusion, daily consumption of quinoa in this short-term intervention appears to modify glucose response, but has minimal effects on other CVD risk biomarkers.

## 1. Introduction

Across the world, cereal-based foods generally form an integral part of the human diet, contributing approximately 30–70% of daily energy [1]. Whole grains, which contain all three anatomical parts of the grain (the bran, germ and endosperm) include the seeds of the common cereals such as rice, wheat, maize and rye as well but they also include the pseudocereals quinoa, amaranth and buckwheat [2]. There is a growing body of epidemiological studies supporting an inverse association between whole grain consumption and risk of several chronic diseases, including cardiovascular disease (CVD), some forms of cancer, type 2 diabetes and obesity, with similar results found across diverse populations [3,4,5,6]. CVD continues to be the leading cause of morbidity and death across the globe, including China, accounting for approximately one third of all deaths [7]. Excess body weight, hypertension and dyslipidemia are clinically considered as the most potent established risk factors for CVD. In China, recent changes to traditional diets, including a dramatic decrease in amounts of whole grains consumed from 104 g/d in 1982 to 24 g/d in 2002 may be responsible for the elevated CVD mortality seen in this country [8].

Quinoa is a member of the *Polygonaceae* family of plant species and it has a similar nutrient composition to grass seeds; since the seed is too small to mill to separate the anatomical fractions quinoa is included in the whole grain category [2]. In addition to a high starchy carbohydrate content, quinoa is a good source of gluten-free protein, which compared with other cereals has a well-balanced amino acid profile; lipids which are rich in unsaturated fats; dietary fibre; and micronutrients and phytochemicals. This supports the use of quinoa as a potential gluten-free alternative to common cereals [9,10,11,12,13].

The effects of quinoa consumption on markers of CVD risk is much less studied either in human or animal studies compared with other whole grains. Although there is some evidence that regular consumption of quinoa-based foods promotes a significant reduction in the concentrations of circulating blood lipids in a few studies, others studies report unclear results, which has led to some discussion about the acute impact of a quinoa-containing diet [14,15,16,17]. In addition to inconsistent results from intervention studies, knowledge of the mechanisms which may cause these observed effects also remains weak, but bioactive components present in quinoa such as dietary fibre, compounds which exhibit high in vitro antioxidant capacity, and other phytochemicals may be the potential contributors [16,18,19,20].

Therefore, the aim of the current study was to evaluate the effect of daily consumption of quinoa on markers of CVD risk, including plasma antioxidant activity, blood glucose, lipids and markers of systemic inflammation, in healthy subjects using a randomized controlled cross-over study.

## 2. Materials and Methods

The study was carried out in accordance with the Declaration of Helsinki; all participants gave written, informed consent before taking part after the study was explained to them verbally. Ethical approval for the study was provided by the University of Newcastle Faculty of Science, Agriculture and Engineering Research Ethics Committee, reference 16-LI-034. Prior to commencement, the study was registered on ClinicalTrials.gov, registration number NCT03036618. Recruitment and interventions were conducted at the NU-Food Food and Consumer Research Facility at the University of Newcastle, UK.

Volunteers were recruited by advertisements using posters and by sending invitation letters to potential volunteers from the local NU-Food volunteer database and by circulating a recruitment email in local organizations. After an initial telephone or email screening on broad criteria to ascertain gender and approximate self-reported body mass index (BMI), recruitment was completed at a screening visit during which the potential volunteers completed an eligibility questionnaire and confirmation of adherence to inclusion/exclusion criteria. Height and weight were measured to calculate BMI as body weight (kg)/height (m^2^). Older, overweight males were selected because they have an elevated risk of CVD compared with younger, normal weight males, but they remain a relatively understudied population. 

A total of thirty-seven healthy overweight and non-smoking male volunteers, aged 36–70 years (mean age 52 years), were recruited for the study which was completed between August 2016 and February 2017. The inclusion criteria were as follows: (1) Healthy males >35 years old; (2) BMI >25 kg/m^2^; (3) Non-smokers with no known previous history of CVD or type 2 diabetes; (4) Not receiving any current medication. Supplement users were included but were asked to stop taking supplements from recruitment for the duration of the study. Individuals were excluded if they reported or were observed to have diabetes or CVD; to have smoked in the past; to have an allergy to gluten, grain products or any ingredients used in the treatment foods; to have experienced recent weight loss (>10%) or plan to lose weight during the study; or to use any medications.

**Figure 1:**
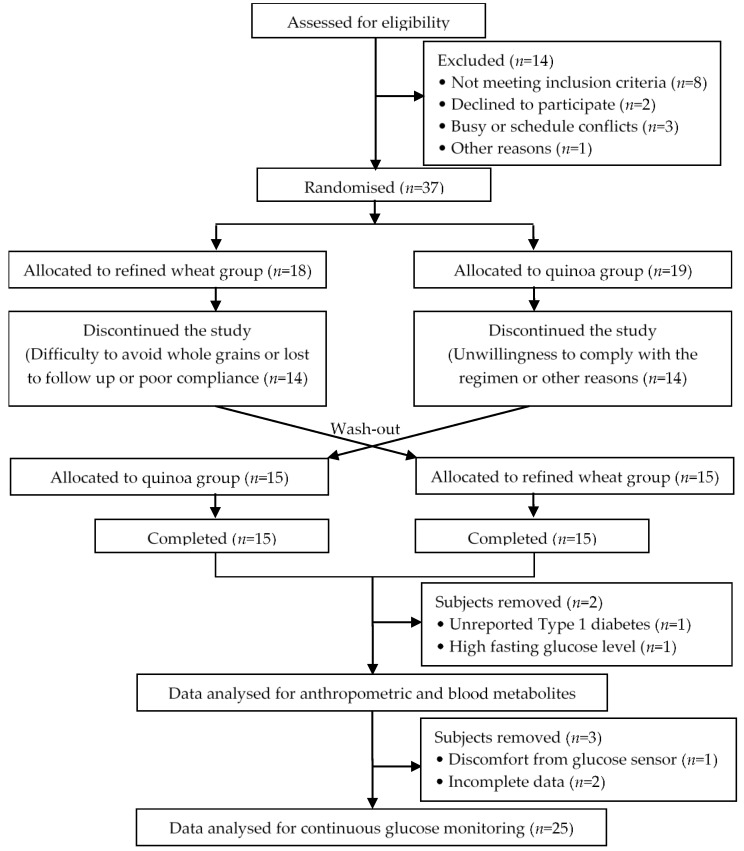
Flow diagram of enrolment, treatment allocation, withdrawals and follow-up of participants through the trial.

Details of study enrolment and completion are shown in Figure 1. In this controlled, cross-over designed study, seven of the 37 subjects randomized to treatments did not complete the study (3 whilst on the quinoa treatment, 4 whilst on the refined wheat treatment, all during the first period of the cross-over study). Reasons for con-completion were difficulty to avoid whole grains (*n* = 2), withdrawn by researcher due to poor compliance (*n* = 1), unwillingness to comply with the regimen (*n* = 1), lost to follow up (*n* = 1), or reasons not given (*n* = 2). A total of 30 men completed the study.

### 2.1. Study Design

**Figure 2:**
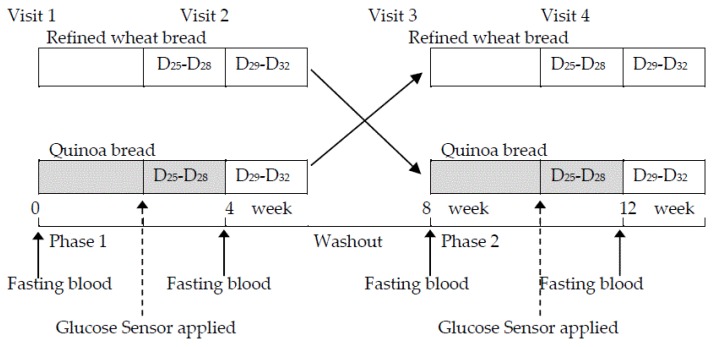
Intervention study design. Randomized controlled crossover study in which subjects received either refined wheat bread or quinoa bread for a period of 4 weeks. Anthropometric and blood pressure measurements were performed and fasting blood samples collected from each volunteer before and after each intervention period as indicated. D_25_–D_28_ last four days of each intervention period; D_29_–D_32_ first four days of the washout period.

The study was a randomized, controlled cross-over trial consisting of two treatment periods of 4 weeks each separated by a washout period of 4 weeks. Subjects were required to attend the NU-Food Facility at University of Newcastle throughout both dietary periods. After the screening, entry into the quinoa treatment arm or refined wheat control arm of the trial was by random allocation using stratification on the basis of age and BMI to ensure that each arm was balanced. During the first 4-week period, one group (*n* = 15) consumed one quinoa roll daily (approximate fresh weight 160 g providing 20 g quinoa flour), and the other group (*n* = 15) consumed a placebo bread (approximate fresh weight 160 g made with 100% refined wheat flour) daily. After the 4-week washout period, subjects who consumed the quinoa bread in the first period crossed over to consume refined wheat bread in the second period, and vice versa (Figure 2). Due to the characteristics of the food products it was not possible to blind the researchers or the subjects during the study to the treatment group/period. However, samples were randomized during analysis and subject codes were not revealed until analysis was complete.

On the first and last day of each intervention period volunteers came to the NU-Food facility after an overnight fast. Body weight was measured in light clothing to the nearest 0.1 kg, as well as body fat percentage by bio-electrical impedance (Tanita BC-420MA). Blood pressure measurements were conducted on the right upper arm with the volunteer in a sitting position after 5–10 min of rest with an automatic Intelli Wrap Cuff (HEM-7321-E, M6 Comfort, Omron Healthcare UK Ltd., Milton Keynes, UK). A fasting blood sample was drawn by venepuncture from the antecubital vein into a vacutainer with EDTA anti-coagulant. Blood samples were mixed and immediately chilled then centrifuged at 1000× *g* at 4 °C. Plasma was removed and stored at −80 °C until analysis.

On visit days at the end of each intervention period, after taking all fasting measurements, a standardized breakfast meal, including 100 g of either the quinoa bread or the 100% refined wheat bread and 10 g strawberry jam, appropriate to the treatment arm they were completing, was served together with ad libitum pure water. The subjects were asked to consume all of the food and to finish the breakfast meal within 3–5 min.

The Abbott FreeStyle Libre Flash Glucose Monitoring (FGM) System (Abbott Laboratories, Maidenhead, Berkshire, UK), including a sensor and reader, is designed to be a continuous monitoring system that does not need finger pricks for calibration, and measures interstitial fluid glucose concentrations. The sensor was applied on to the back of the upper arm of subjects using the applicator by trained staff on day-23 or day-24 and was worn up until after day-32. The sensor records glucose concentrations every 15 min providing comprehensive data for a complete glycaemic profile of the wearer. The sensor stores the recorded data which is downloaded to a monitor/reader by the wearer. To obtain a complete glycemic picture, the sensor must be scanned at least once every eight hours, and the reader can capture data when it is within 1 cm to 4 cm of the sensor. With every scan the reader also provides a current glucose reading, together with (up to) the last 8 h of glucose data and a trend arrow showing the direction that the blood glucose values are heading. Cumulative data for the period the sensor was worn by the volunteer was downloaded to a PC in a spreadsheet format for analysis.

### 2.2. Study Food

The refined wheat and quinoa bread used in this study were manufactured by one bakery (The Artisan Baking Community, Earth Doctors Ltd., Northumberland, UK). For quinoa-containing bread, 20% of the refined wheat flour was replaced with quinoa flour with the rest of the ingredients remaining exactly the same. The 20% inclusion rate was chosen on the basis of sensory analysis of breads containing 20% or 30% quinoa flour as the lower inclusion rate was deemed more acceptable and could be consumed readily by participants (data not shown). The breads were baked as rolls, each providing 100 g of flour; each roll weighed approximately 160 g after baking. Freshly baked bread were packaged in single-serving food & freezer bags and stored frozen at −20 °C before dispensing to volunteers.

The subjects were asked to keep the bread in their home freezers and were instructed to take them out of the freezer and ensure they were fully defrosted before consuming. To ensure that the subjects ate the correct amounts of test bread, they were advised to consume one roll daily, in place of other bread products (e.g., standard bread, pitas, bagels, dinner bread) or carbohydrate-rich foods (e.g., rice, pasta), in their diet. They were advised to eat the bread with their regular portion of different products, such as spreads, cheese, jam and salad. During the 13-week trial, subjects were instructed to avoid consuming any other source of whole grains or related products derived from whole grains, such as bran flakes, wholemeal bread and brown rice (a list was provided), and they were also asked not to take any vitamin or mineral supplements during the intervention. In addition, the participants were asked not to change their normal consumption of the following foods that potentially change markers of CVD risk: coffee, tea, oily fish and dark chocolate. A list of foods that could be freely consumed was also offered with instructions to maintain their normal physical activity, dietary and lifestyle patterns while on the study.

Compliance with the diet was checked by 3-day food records and daily records. Three-day food records including two weekdays and one weekend day were collected from the subjects before and at the end of each treatment arm. Additionally, during the intervention periods, the subjects were requested to report if they had experienced any possible side effects related to the study bread, such as flatulence, stomach problems, abdominal or bloating pain. Also, a food frequency questionnaire (FFQ), which recorded food intake during the previous 7 days only, was undertaken by each subject before and at the end of each intervention period. The FFQ also contained questions regarding dietary habits including how much and how often the participants consumed vegetables, eggs, milk and meat, etc.

### 2.3. Analytical Methods

#### 2.3.1. Compositional Analysis of Refined Wheat and Quinoa Roll

After drying in the oven at 90 °C overnight, refined wheat and quinoa bread were ground to flour (Sieve size, 1 mm) using a laboratory cyclone mill twister (Retsch, Haan, Germany), then packed in grip seal bags and stored in −20 °C freezer until use. The free, conjugated and bound phenolic compounds in refined wheat and quinoa roll flours were isolated according to the previous method with slight modifications [21,22]. Total phenolic content of refined wheat and quinoa roll extracts were determined using the Folin-Ciocalteu method as described by Zhang and colleagues with minor modifications [23]. 

Refined wheat and quinoa bread were analysed for total energy, protein (Dumas method), ash (BS 4401-1:1998), moisture (BS 4401-3:1997), total sugars (Ion Chromatography), sodium (ISO 7485:2000), salt (calculated from sodium), insoluble dietary fibre (AOAC 991.43), soluble dietary fibre (AOAC 991.43), total dietary fibre (AOAC 991.43), fat (based on BS 4401-4:1970), saturated fat (ISO 12966-2:2011), monounsaturated fat (ISO 12966-2:2011) and polyunsaturated fat (ISO 12966-2:2011). Available carbohydrate content was calculated by difference from the formula: available carbohydrates = 100 − (protein + ash + moisture+ dietary fibre + fat). All of the above analyses were carried out to British Standards by an accredited company (Alex Stewart Agriculture Ltd., Liverpool, UK). The amino acid profile of the breads was determined by HPLC after acid hydrolysis by an accredited company (AM/V/206, ALS Food and Pharmaceutical, Mirfield, UK).

#### 2.3.2. Plasma and Urine

Blood plasma metabolites were analysed using an ABX Pentra 400 (Horiba Medical, Northampton, UK), using the following standard enzymic procedures (reference number, procedure): glucose (A11A01668, Peroxidase), total cholesterol (A11A1634, Cholesterol esterase/Cholesterol Oxidase), low-density lipoprotein (LDL) cholesterol (A11A01638, Detergent/Cholesterol Oxidase/Esterase), high-density lipoprotein (HDL) cholesterol (A11A01636, Polyanions), Triglycerides (A11A01640, p-Chlorophenol + 4-aminoantipyrine), Apo A1 (A11A01687, Turbidometric Immunoassay), Apo B (A11A01688, Turbidometric Immunoassay), C-reactive protein (CRP) (A11A01611, Latex Turbidometric Immunoassay), Aspartate transaminase (AST) (A11A01629, International Federation of Clinical Chemistry), Alanine transaminase (ALT) (A11A01627, International Federation of Clinical Chemistry). Plasma insulin was determined by enzyme-linked immunosorbent assay (ELISA) using the Invitrogen Human Insulin (KAQ 1251) kit (Invitrogen, ThermoFisher Scientific, Waltham, MA, USA). Plasma free fatty acids (NEFAs) were determined by the Acyl-CoA synthetase Acyl-CoA oxidase (ACS-ACOD) method using the Wako NEFA-HR(2) kit (Wako Chemicals GmbH, Neuss, Germany). Plasma antioxidant activity was determined using the FRAP and TEAC assays as described above.

### 2.4. Sample Size

Sample size was determined using a Paired *t* Test approach in Statistical Software Minitab 17, based on LDL cholesterol concentration data reported in the two papers describing quinoa interventions by Farinazzi-Machado and colleagues (mean baseline ± standard deviation: 3.95 ± 1.03 mmol/L; observed changes: −20.53%; *p*-value = 0.0002) and De Carvalho and colleagues (mean baseline ± standard deviation: 3.35 ± 0.92 mmol/L; observed changes: −5.87%; *p*-value <0.05) that used a similar quinoa dosage, as well as a recent dietary intervention study completed in Newcastle UK [15,16]. Using levels of significance of less than 0.05 and 80% power, a sample size of between ten and twelve subjects per group was required to observe a 10% decrease in LDL cholesterol concentration in the intervention group, assuming that no change would occur in cholesterol concentration in the control group. To account for dropout, a minimum of sixteen subjects per group were targeted for recruitment.

### 2.5. Calculations and Statistical Analyses

The 3-day food records were analysed by Windiets 2015 (Robert Gordon University, Aberdeen, UK). The biochemical and dietary data were analysed by using the SPSS 22.0 for Windows statistical program (SPSS Inc., Chicago, IL, USA). The results are expressed as means and standard deviations. Normality of the variables was checked with the Shapiro–Wilk test, and data that were not normally distributed were transformed (using the log10 function) prior to statistical analyses and then back-transformed for presentation of results. To determine the differences in the measured variables following intake of the refined wheat and quinoa bread, a paired *t*-test was used with *p* < 0.05 considered to be significantly different. The percentage change was calculated as follows: (value at 4 week − value at baseline)/value at baseline × 100. 

The area under the continuous glucose response curve (AUC) was determined for blood glucose determined from the glucose monitor using the trapezoid method in GraphPad Prism (version 7.01; San Diego, CA, USA), and the results expressed as means and standard deviations. Data were analysed for the last 4 days (D_25_–D_28_) of each intervention period, the following 4 days in the washout periods (D_29_–D_32_), the total of these 8 days (D_25_–D_32_) for the two treatment periods after the sensor had been applied, and the time periods 0–240 min after the test breakfast at the end of each treatment period (for comparison of results from the glucose monitor and the protein saver card). The changes within refined wheat or quinoa treatment in the AUC for glucose were expressed as a percentage of initial AUC for glucose: changes (%) = ((D_29_–D_32_) − (D_25_–D_28_))/(D_25_–D_28_) × 100.

## 3. Results

### 3.1. Composition of 100% Refined Wheat and 20% Quinoa Bread

The results for compositional analysis are shown in Table 1. The composition of the two test breads was broadly very similar, with a slightly higher protein and lower available carbohydrate content in the quinoa bread compared with the refined wheat bread. The fibre content of the quinoa bread was higher than the refined wheat bread mainly due to a higher content of insoluble fibre. The phenolic content of the quinoa bread was slightly higher than the refined wheat bread (Table 1). The amino acid profile of the two breads is shown in Table 2.

### 3.2. Anthropometric Variables

Anthropometric data for the study participants are shown in Table 3. The mean age of participants was 51.54 years (range from 36 to 70). Average BMI at baseline was 27.7 kg/m^2^. Mean body weight, BMI and body fat percentage did not change throughout the study period. Systolic (SBP) and diastolic (DBP) blood pressure values were mildly elevated (SBP > 120 mmHg) but did not change during the intervention period with either of the treatments (*p* > 0.05).

### 3.3. Blood Variables and Antioxidant Capacity

At the end of the intervention, there were no significant effects of quinoa and refined wheat bread consumption on fasting plasma concentrations of insulin, total cholesterol, HDL cholesterol, NEFAs, ApoA1, ApoB, AST, ALT and CRP with respect to corresponding baseline measurements (Table 3). The percentage change (Δ%) from baseline was not different between interventions for any of the parameters measured. Neither treatment affected the ratios of HDL/Total cholesterol or ApoB/ApoA1. However, after 4 weeks of quinoa consumption, there was a significant decrease in glucose by 4.5% and LDL cholesterol by 5.7% compared with the corresponding baseline, but the percentage change from baseline between the two treatments did not reach significance. There was an unexpected increase in triglycerides concentration by 14.3% after 4 weeks of consuming the quinoa bread (*p* = 0.049), with no difference after wheat consumption, although there was no significant difference in the percentage change between the two treatments. Plasma antioxidant capacity analysed by both FRAP and TEAC assays was not affected by the treatments.

### 3.4. Dietary Intake and Compliance

Nutrient intake was not affected during the intervention study except for an apparent increase in carbohydrate intake during the refined wheat bread period (Table 4). Self-reported compliance from daily records was very good. Both breads were well tolerated by the subjects, without any apparent side effects or complaints.

### 3.5. Continuous Glucose Monitoring

Of 28 subjects with sensor records, one subject removed the sensor early due to discomfort, and three subjects had less than 4 days of data because of accidental removal or poor compliance with transferring data to the reader. These subjects were subsequently removed from analysis, thus, full sensor records on 24 subjects remained for analysis (primary sensor failure of 14.3%).

#### 3.5.1. Area under Curve Glucose Response Curve

**Figure 3:**
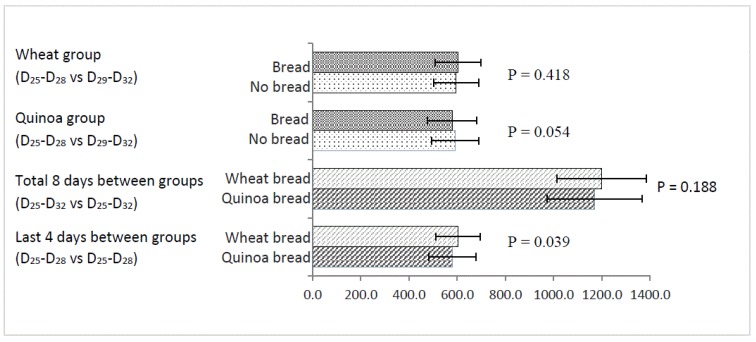
The area under curve of glucose concentration responses to intake of refined wheat and quinoa bread during 8 days (D_25_–D_32_). Values are means with their standard deviations represented by horizontal bars (*n* = 24). Bread means 4-day period whilst consuming the study breads at the end of the treatment period (D_25_–D_28_); No Bread means the first 4 days of the washout period (D_29_–D_32_). Difference was considered as significant if *p* < 0.05 (paired-samples *t* test).

Data for AUC the glucose response curve determined from the continuous glucose monitors are shown in Figure 3. There was no effect on glucose AUC between subjects during the supplementation period (D_25_–D_28_) when consuming refined wheat bread or quinoa bread or in the early wash-out period (D_29_–D_32_). However, there was a trend for an increase in glucose AUC by 2.0% for the first 4 wash-out days after quinoa bread consumption compared with the last four days of quinoa consumption (*p* = 0.054). Moreover, there were significant difference between treatments in changes in the AUC for glucose when expressed as a percentage of initial AUC for glucose ((glucose AUC in wash-out period (D_29_–D_32_)---glucose AUC in refined wheat bread or quinoa bread period (D_25_–D_28_))/glucose AUC in refined wheat bread or quinoa bread period (D_25_–D_28_) × 100: refined wheat bread −2.2%, quinoa bread 2.0%, *p* < 0.001). This reflects the lower AUC during the quinoa treatment compared with the wheat treatment and the early washout phase. There was no statistical difference in AUC for glucose for the total 8 days between treatments (D_25_–D_32_ W vs. D_25_–D_32_ Q) although it was numerically higher in the wheat treatment period. However, the AUC for glucose during the last 4 days (D_25_–D_28_) of the quinoa treatment was significantly lower than that for the same period of the refined wheat treatment (*p* = 0.039). 

#### 3.5.2. Postprandial Glucose

**Figure 4:**
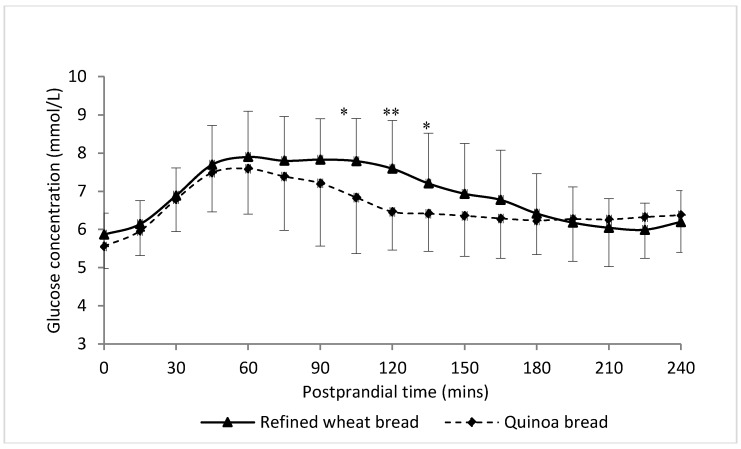
Mean concentrations of glucose responses for 240 min after intake of refined wheat bread and quinoa bread meals after 4 weeks of intervention measured by continuous glucose monitoring. Values are means with their standard deviations represented by vertical bars (*n* = 24 for refined wheat bread treatment and quinoa bread treatment). * Mean values were significantly different between meals: * *p* < 0.05, ** *p* < 0.01 (paired-samples *t* test with Bonferroni correction).

The measurements of postprandial glucose concentrations derived from the continuous glucose monitors over the 4 h postprandial period are shown in Figure 4. No effects of the order of quinoa or refined wheat bread consumption were detected in glucose responses. At baseline, the glucose concentration was slightly lower in the quinoa treatment than in the wheat treatment but this was not statistically significant (*p* > 0.05). After the test meals plasma glucose concentrations increased significantly from the baseline concentration for both treatments, and remained above baseline up to 4 h post-meal. Following the quinoa roll treatment, the glucose responses at 105, 120 and 135 min were significantly lower than those after the control meal (*p* < 0.05, *p* < 0.01 and *p* < 0.05, respectively). The AUC for glucose for the 4 h glucose responses was, on average, 5.6% (*p* < 0.05) lower after consumption of the quinoa bread compared with the control meal. The glucose response curve following the refined wheat meal was at its highest at about 60 min and remained at approximately the same concentration up to 120 min. The peak glucose concentration was also observed at 60 min after the ingestion of the quinoa meal, but then decreased more rapidly until 120 min and then more slowly between 120 and 240 min. The difference in peak glucose response or time to peak glucose response on the two meals did not reach statistical significance (*p* = 0.177 and *p* = 0.235, respectively) due to highly variability in individual responses.

## 4. Discussion

Whole grain cereals are recognized as an important part of a healthy diet, and at least 3 to 5 servings (16 g/serving) daily are recommended in the 2015–2020 US dietary guidelines [24]. Similarly, the 2013 Australian Dietary Guidelines recommend that adults aged 19–50 should consume at least 6 servings of grain per day of which two thirds should be whole grain; the Australian guidelines also specifically identify quinoa in the cereal group [25]. Epidemiological studies consistently suggest an inverse association between the intake of whole-grain food and risk of CVD, type 2 diabetes, cancer and obesity [4,26].

Quinoa, as a whole grain, has attracted considerable attention recently, yet little research has been done on its beneficial effects against CVD risk. To our knowledge only four quinoa intervention studies in humans have been published and these describe variable results [14,15,16,17]. The present study assessed changes in CVD risk markers in response to daily intake of quinoa in the form of an enriched bread delivering 20 g quinoa per day, compared with refined wheat bread over a 4-week period, using a randomised crossover experimental design. The results showed that intake of quinoa bread failed to produce any favorable changes in metabolic variables compared with refined wheat bread. Although there was a reduction in LDL cholesterol (−5.7%) in the present study between baseline and after consuming quinoa which was comparable to the significant reduction (−5.9%) in the study of De Carvalho and colleagues [16]; the absence of differences between treatments in the present study was due to an unexpected cholesterol-lowering effect after the refined wheat bread treatment. The lack of improvement in fasting plasma lipids in the present study is in agreement with the study of Zevallos and colleagues which analysed the effects of consuming 50 g quinoa per day for 6 weeks as part of their usual diets in celiac patients, as well as the study by Jenkins and colleagues except for significantly increased HDL cholesterol in the latter study [14,17]. However, this finding is in contrast with two other studies using similar doses and duration by Farinazzi-Machado and colleagues and De Carvalho and colleagues, in which total, LDL cholesterol and triglycerides but not HDL cholesterol levels were significantly lowered after approximately 1 month of consuming 19.5 g quinoa bars and 25 g quinoa flakes in healthy students and overweight postmenopausal women, respectively [15,16].

Anthropometric and blood variables did not significantly differ between treatments at study entry, and dietary intake data suggested that the participants’ habitual diet and energy intake were not affected by the intervention. Body weight, BMI and body fat content were stable throughout the study. The dose of whole grain quinoa used in the study was probably too small to change anthropometric variables which has been seen in some whole grain interventions which have used higher doses and were based on whole-grain wheat, rye or oats compared with refined alternatives [27,28,29].

Postprandial hyperglycemia has been recognised as a risk factor for CVD, resulting in elevated cardiovascular morbidity and mortality in diabetic subjects [30]. There has been one human intervention study which showed a significant reduction in fasting plasma glucose concentration after inclusion of two slices of quinoa bread into each individual’s habitual diet for 6 months. However, this study was performed in 210 patients with type 2 diabetes, and although quinoa was included in the intervention it was only part of a wider dietary changes [14].

In the present study, the effects of a breakfast meal consisting of the test quinoa and wheat bread on acute glycemic responses was investigated using continuous glucose monitoring in overweight but otherwise healthy subjects. The glucose response curves were relatively similar but were consistently lower after the quinoa bread compared with the refined wheat bread (Figure 4). These results are in agreement with those of Gabrial and colleagues, showing a reduced capillary blood glucose response in healthy subjects after consuming 80 g quinoa compared with white wheat bread as a reference [31]. In this earlier study, the quinoa meal resulted in significantly lower AUC values for blood glucose in diabetic subjects compared with the white wheat bread, while no significant difference was detected in healthy subjects. The differences in the postprandial glucose responses in the present study are unlikely to be explained by the time taken to eat the breakfast, since the subjects were observed and encouraged to finish the meals in 3–5 min. Several previous in vitro and in vivo studies have shown that polyphenols may affect carbohydrate digestion and absorption and thereby postprandial glucose responses [32,33,34,35]. For example, delayed absorption of glucose after intake of coffee and apple juice by humans has been demonstrated [36,37]. However, the additional polyphenolics derived from the low dose of quinoa incorporated in the breakfast meal may only have negligible effects on the postprandial glycaemia observed. In addition to providing more polyphenols, the higher insoluble fibre content (1.33 and 2.84 g for the refined wheat and quinoa bread, respectively) may also affect glucose response although the overall contribution to the meal, and the whole diet was still relatively small. Insoluble fibre consumption increases the rate of small intestine transit, thus, resulting in reduced starch hydrolysis and absorption [38,39].

Interestingly, the results of chronic continuous glucose monitoring suggest that regular consumption of quinoa resulted in a significantly lower area under the glucose response curve for the last 4 days of the quinoa intervention period compared with the same period during the refined wheat bread intervention (Figure 3). The area under the glucose response curve also tended to be lower at the end of the quinoa intervention compared with the wash-out period (*p* = 0.054). This suggests that quinoa in the diet may reduce glucose uptake from the diet. However, an important limitation of this investigation was that the amounts of available carbohydrate and other macronutrients were not equalized between treatments. As a result, the quinoa bread was slightly lower in available carbohydrate in comparison with the wheat bread, and may result in a lower glycemic response. There was also a significant increase in total carbohydrate intake during the refined wheat intervention period which was unexplained and may have influenced glucoseresponse. This requires further investigation with a better control of dietary intake.

It has been suggested that inflammation is an important contributor to the development of atherosclerosis. Plasma high-sensitivity CRP (hsCRP) is frequently used as a marker of inflammation; in this study we measured CRP which is less specific than hsCRP and this did not change during the intervention, nor the study by Jenkins and colleagues [14], although in our study values for participants were all within the normal range and might not be expected to change. The hepatic enzymes AST and ALT are sensitive indicators of liver damage or injury from various diseases or conditions. In the present study, no significant changes in AST and ALT levels were detected after quinoa consumption, although ALT concentrations were numerically decreased, in contrast to the study of Farinazzi-Machado and colleagues which reported lower values of AST, with ALT unaffected after 1 month consumption of quinoa bars [15]. In an animal study, the mean values of serum AST and ALT were significantly reduced in male Wistar strain albino rats fed with a high fat diet with 60% milled quinoa [40]. This was attributed to the high levels of quinoa polyphenols, but this has not been confirmed in human subjects to date.

Overall, the results from this study and the four previous intervention studies mentioned above should be viewed or explained with caution due to inappropriate (non-refined grain) or lack of control treatments used in some of the studies. Difference in subject characteristics, study duration, amounts and mode of quinoa foods provided in studies may account for the variable results. For example, the study duration represents a short period of dietary alteration in the context of lifelong dietary exposures, and may not have been sufficiently long to detect favourable effects on markers of CVD risk. The lack of intervention effects may also be a result of the low dose of quinoa consumed in this study. According to epidemiological studies, beneficial health effects of whole grains can be expected at a level of 3 servings (48 g) per day [4,41]. The daily amounts of quinoa provided during the present study (20 g) was in the range of usual whole grain intake of about 16–25 g/day, but much lower than minimum recommended intake of 48–96 g/day [42]. In this study, only refined wheat and wheat-quinoa breads were provided for inclusion in the participants’ usual diets. The nutrient content of the two breads was similar, with the exception of a higher concentration of total dietary fibre in quinoa bread, as expected and a higher intake of polyphenolics (Table 1). Many epidemiological studies have shown that soluble fibre was the main contributor to the cholesterol-lowering property of whole grains [43,44]. However, the higher content of insoluble dietary fibre in the quinoa bread was the main cause of the large difference in total dietary fibre between the two breads. This may in part explain the lack of the response in plasma cholesterol in the current study. Higher doses of polyphenolics and polyunsaturated fatty acids have been suggested to have cardio-protective effects in humans, but it is unlikely that these levels of intake could be achieved without much higher amounts of quinoa in the diet than that used in the present study [45,46]. In this context the effects of higher doses of quinoa would be interesting to observe.

## 5. Conclusions

The results of the dietary intervention study indicate that consumption of 20 g quinoa per day in the form of a wheat-quinoa bread roll does not affect markers of CVD risk, although there is a suggestion that glycaemia may be improved through a reduction in postprandial glycemic response. The primary outcome of the trial, change in LDL-cholesterol concentration was not significantly affected by the intervention. To the best of our knowledge, this is the first study analysing the effects of food intake in a short-term intervention study on glucose concentrations derived from FGM to date, which showed significantly lower AUC for glucose over the four days at the end of the quinoa treatment period than that for the same period of the wheat treatment. Overall the results suggest some potential benefit of consuming quinoa on glucose response but this requires further investigation, possibly with larger doses of quinoa. The mechanisms by why quinoa may have this effect remain undetermined.

## Figures and Tables

**Table 1:** Proximate composition of refined wheat and quinoa bred (dry basis) ^1^.

Nutrient	100% Refined Wheat Bread	20% Quinoa Bread
Energy (kcal/100 g)	381	381
(kJ/100 g)	1615	1612
Protein (g/100 g)	12.63	14.04
Ash (g/100 g)	2.37	2.87
Moisture (g/100 g)	2.69	3.02
Available Carbohydrate (g/100 g)	77.05	72.64
Total sugars (g/100 g)	6.67	5.78
Sodium (g/100 g)	0.51	0.62
Salt (g/100 g)	1.30	1.58
Insoluble dietary fibre (g/100 g)	2.17	4.99
Soluble dietary fibre (g/100 g)	1.43	1.53
Total dietary fibre (g/100 g)	3.60	6.52
Fat (g/100 g)	1.66	2.73
Saturated fat (g/100 g)	0.29	0.37
Monounsaturated fat (g/100 g)	0.24	0.68
Polyunsaturated fat (g/100 g)	1.06	1.56
Total phenolic content (mg GAE/g) ^2^		
Free	0.52 ± 0.04	0.76 ± 0.06
Conjugated	0.08 ± 0.01	0.16 ± 0.01
Bound	0.08 ± 0.01	0.19 ± 0.02
Total	0.77	1.11

^1^ Each roll contained 100g dry matter and 1 roll was consumed per day; ^2^ mg gallic acid equivalents/g; mean of 3 replicates ± standard deviation (SD).

**Table 2:** Composition of amino acid of refined wheat and quinoa rolls (dry basis) ^1^.

Amino acid	100% Refined Wheat Bread	20% Quinoa Bread
Essential amino acids		
Histidine (g/100 g)	0.28	0.35
Iso-Leucine (g/100 g)	0.49	0.46
Leucine (g/100 g)	0.90	0.78
Lysine (g/100 g)	0.32	0.81
Methionine (g/100 g)	0.18	0.27
Phenylalanine (g/100 g)	0.66	0.48
Threonine (g/100 g)	0.38	0.50
Valine (g/100 g)	0.59	0.59
Non-essential amino acids		
Alanine (g/100 g)	0.41	0.58
Arginine (g/100 g)	0.53	1.07
Aspartic Acid (g/100 g)	0.56	1.07
Cystine (g/100 g)	0.29	0.20
Glutamic Acid (g/100 g)	4.55	1.70
Glycine (g/100 g)	0.49	0.68
Proline (g/100 g)	1.56	0.49
Serine (g/100 g)	0.74	0.62
Tyrosine (g/100 g)	0.39	0.39

^1^Each roll contained 100g dry matter and 1 roll was consumed per day.

**Table 3:** Effect of refined wheat and quinoa bread on anthropometry, blood pressure, blood parameters and plasma antioxidant capacity measures.

Outcome	Refined Wheat Bread (*n* = 28)					Quinoa Bread (*n* = 28)					
	Baseline		Week 4			Baseline		Week 4			*p* for
	Mean	SD	Mean	SD	Δ% ^2^	Mean	SD	Mean	SD	Δ%	Change ^3^
**Anthropometric variables ^1^**											
Age	51.5	10.7	-	-	-	-	-	-	-	-	-
Body weight (kg)	85.8	9.5	86.0	9.3	0.2	85.9	9.6	85.8	9.5	−0.1	0.279
BMI (kg/m^2^)	27.6	2.3	27.7	2.3	0.3	27.7	2.4	27.6	2.4	−0.4	0.281
Body fat percentage (%)	25.4	5.2	25.7	5.0	1.2	25.4	5.2	25.2	5.0	−0.8	0.331
Blood pressure (mmHg)											
Systolic	129.5	12.2	130.5	12.2	0.8	128.8	12.6	128.5	11.0	−0.2	0.272
Diastolic	85.4	10.2	86.4	14.5	1.2	84.1	9.7	85.8	10.1	2.0	0.726
**Blood variables**											
Glucose (mmol/L)	5.71	0.56	5.64	0.53	−1.2	5.84	0.63	5.58	0.68	−4.5 **	0.103
Insulin (pmol/L)	54.9	21.09	58.4	26.67	6.5	54.1	25.4	61.5	32.32	13.7	0.278
Cholesterol (mmol/L)											
Total	5.63	1.20	5.50	0.99	−2.3	5.64	1.04	5.54	0.78	−1.8	0.769
LDL	3.53	0.93	3.36	0.76	−4.8	3.49	0.84	3.29	0.65	−5.7 *	0.439
HDL	1.36	0.25	1.38	0.23	2.0	1.39	0.22	1.39	0.22	0.2	0.363
Triglyceride (mmol/L)	1.41	0.59	1.53	0.59	8.9	1.39	0.52	1.59	0.57	14.3 *	0.587
NEFAs (mmol/L)	0.39	0.17	0.36	0.14	−9.9	0.42	0.17	0.35	0.15	−16.2	0.496
Apo (g/L)											
ApoA1	1.38	0.19	1.38	0.18	0.0	1.41	0.18	1.39	0.16	−1.5	0.415
ApoB	1.16	0.25	1.12	0.20	−3.3	1.15	0.21	1.12	0.16	−2.1	0.798
Ratios											
HDL/Total	0.25	0.08	0.26	0.05	7.0	0.25	0.06	0.26	0.05	3.2	0.295
ApoB/ApoA1	1.25	0.36	1.27	0.27	3.5	1.26	0.26	1.26	0.26	0.4	0.203
CRP (mg/L)	1.69	0.71	2.21	0.69	30.8	1.72	0.41	1.43	0.28	16.9	0.197
AST (U/L)	41.1	17.79	40.2	16.55	−2.1	47.2	47.4	42.8	27.97	−9.3	0.526
ALT (U/L)	34.5	26.80	35.7	26.39	3.4	60.4	46.8	39.3	38.61	−34.9	0.277

^1^ BMI, body mass index; CRP, C-reactive protein; AST, aspartate transaminase; ALT, alanine transaminase. Mean values were significantly different from respective baseline: * *p* < 0.05; ** *p* < 0.01. ^2^ percentage change from respective baseline. ^3^ *p* for difference in change for wheat bread compared with quinoa bread.

**Table 4:** Mean daily intake of nutrients before and at the end of each treatment arm in all subjects, mean values ± SD.

Nutrient	Refined Wheat Bread (*n* = 28)			Quinoa Bread (*n* = 28)			
	Baseline	Week 4	Δ% ^1^	Baseline	Week 4	Δ%	*p* for Change ^2^
Energy (kJ)	9505 ± 1320	10,065 ± 1275	5.9	9600 ± 1630	9956 ± 1685	3.7	0.733
Carbohydrates							
(g/day)	241 ± 50	285 ± 46	18.3 *	250 ± 60	270 ± 43.1	8.0	0.623
(%E)	50.2 ± 6.1	53.1 ± 5.0	5.8	49.5 ± 7.9	52.2 ± 5.5	5.5	0.799
Protein							
(g/day)	79.2 ± 16.1	87.5 ± 20.3	10.5	81.5 ± 16.9	90.4 ± 17.4	10.9	0.347
(%E)	17.0 ± 2.2	15.8 ± 2.4	−7.1	15.7 ± 2.2	16.9 ± 3.5	7.6	0.414
Fat							
(g/day)	64.2 ± 13.1	71.0 ± 18.4	10.6	76.5 ± 20.1	72.2 ± 19.8	−5.6	0.424
(%E)	29.8 ± 5.2	28.5 ± 4.5	−4.4	32.6 ± 5.0	28.9 ± 4.2	−11.3	0.396
SFA							
(g/day)	27.5 ± 5.4	29.3 ± 7.9	6.5	32.8 ± 8.9	30.7 ± 9.4	−6.4	0.198
(%E)	12.8 ± 2.2	12.0 ± 1.9	−6.3	13.9 ± 2.7	12.6 ± 2.2	−9.4	0.465
MUFA							
(g/day)	23.4 ± 5.8	25.7 ± 6.6	9.8	27.5 ± 6.8	25.0 ±7.6	−9.1	0.785
(%E)	10.9 ± 2.0	10.2 ± 1.5	−6.4	11.6 ± 2.1	10.2 ± 1.4	−12.1	0.743
PUFA							
(g/day)	9.3 ± 2.5	10.7 ± 2.4	15.1	10.3 ± 2.8	10.3 ± 2.4	0	0.854
(%E)	4.5 ± 0.8	4.4 ± 1.0	2.2	4.8 ± 0.7	4.3 ± 1.0	−10.4	0.782
Alcohol							
(g/day)	5.3 ± 2.3	6.6 ± 4.0	24.5	4.5 ± 1.9	5.2 ± 2.8	15.5	0.587
(%E)	1.8 ± 0.8	2.2 ± 1.3	22.2	1.5 ± 0.7	1.5 ± 0.9	0	0.154
Dietary fibre (g/day)	22.1 ± 5.6	20.1 ± 6.4	−9.0	24.7 ± 6.9	22.5 ± 4.8	−8.9	0.687

Mean values were significantly different from respective baseline (week 0): * *p* < 0.05. ^1^ percentage change from respective baseline. ^2^ *p* for difference in change for wheat bread compared with quinoa bread.
